# Supplementary material for: Folding Features and Dynamics of 3D Genome Architecture in Plant Fungal Pathogens
Source: Microbiol Spectr. 2022 Oct 17;10(6):e02608-22. doi: 10.1128/spectrum.02608-22 (PMC9769607; doi:10.1128/spectrum.02608-22)
Supplement: Supplemental file 1 — Fig. S1 to S10. Download spectrum.02608-22-s0001.pdf, PDF file, 6.0 MB [file spectrum.02608-22-s0001.pdf]

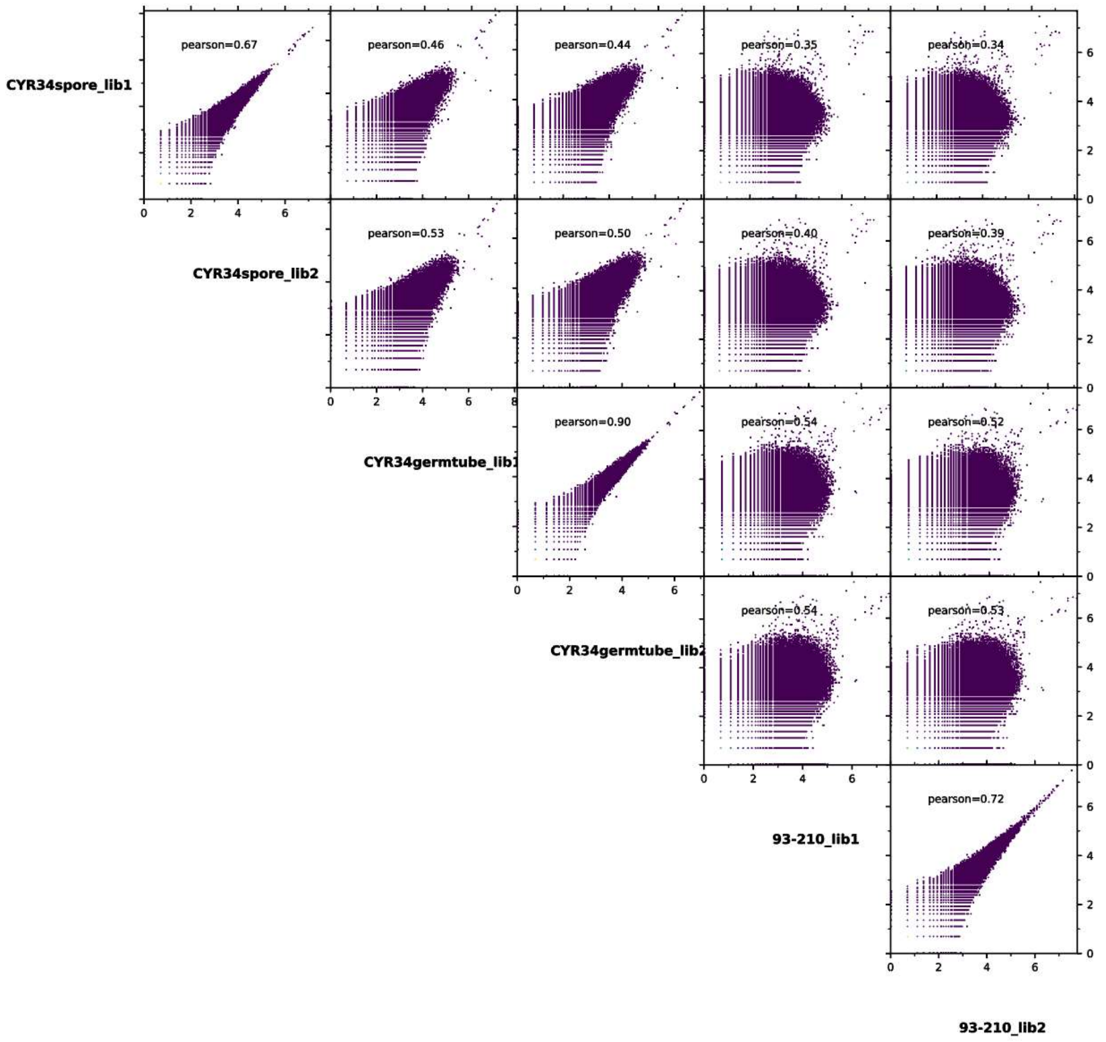

Extended Data Fig S1. Pairwise scatter plot of correlations of Hi-C sequencing libraries generated in this study. Note: the high Pearson correlation values between the libraries from the same sample, and the low Pearson correlation values between libraries from different samples, indicating the high quality and reproducibility of our Hi-C sequencing libraries.

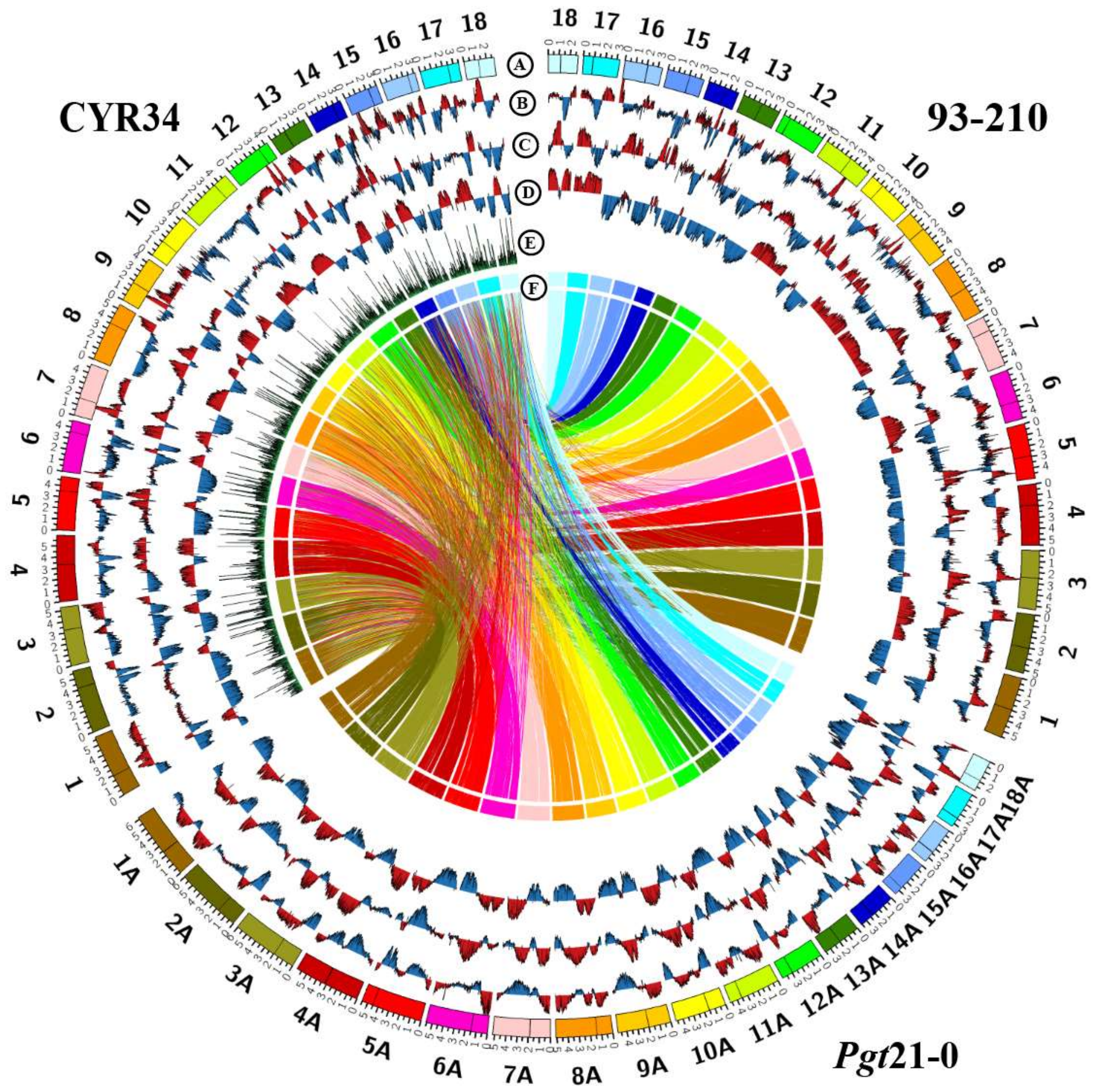

Extended Data Fig S2. Circos plot of collinearity among CYR34, 93-210 and *Pgt*210 genomes and PCA eigenvectors for Hi-C matrices. Track Ⓐ: chromosome segments; track Ⓑ: PC3; track Ⓒ: PC2; track Ⓓ: PC1; track Ⓔ: abundance of gene expression (in FPKM) in CYR34 urediniospore, data from other isolates were not available; track Ⓕ: synteny relationship among CYR34, 93-210 and *Pgt*210 genomes, each colored line links a pair of homologous genes.

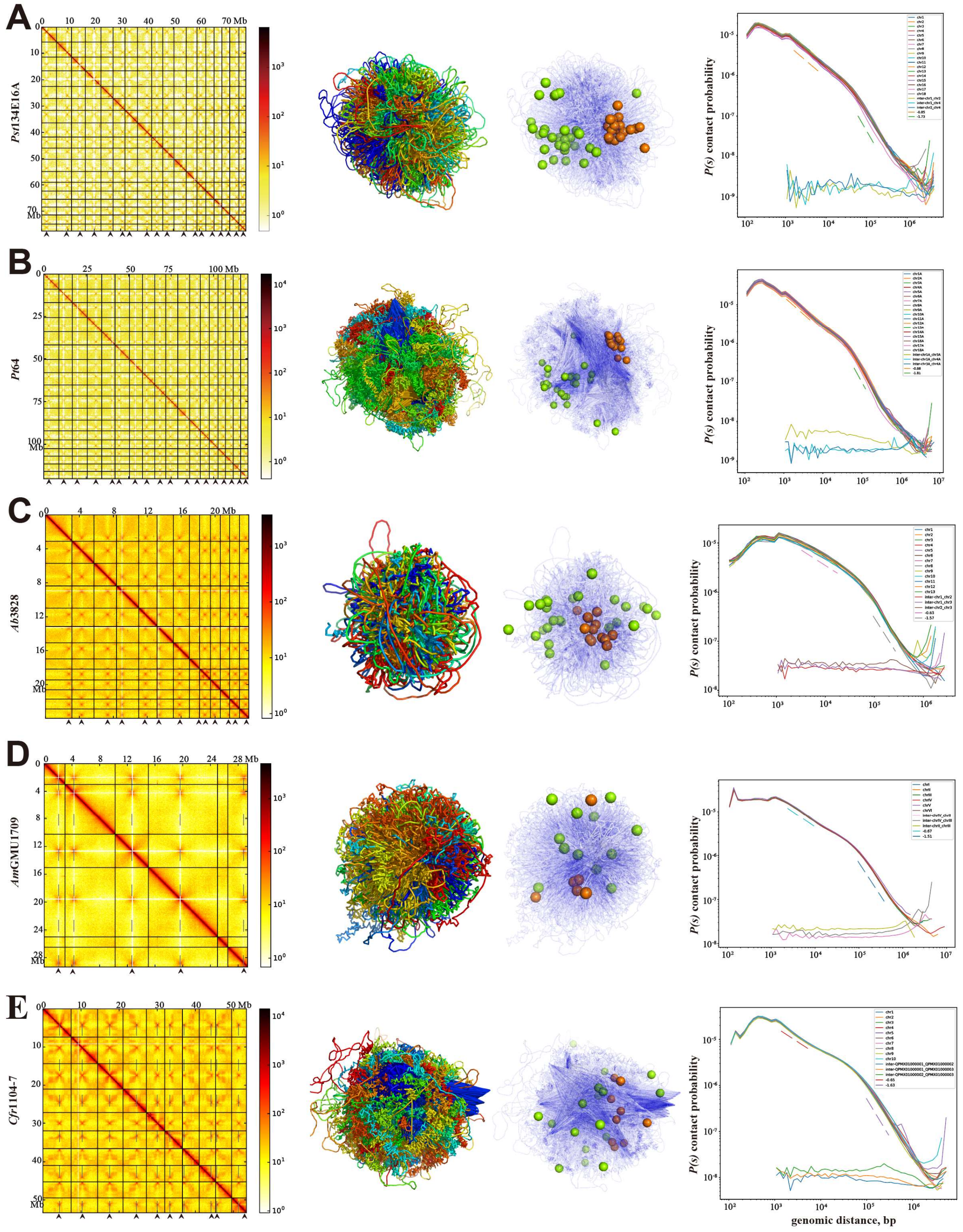

Extended Data Fig S3. *to be continued*

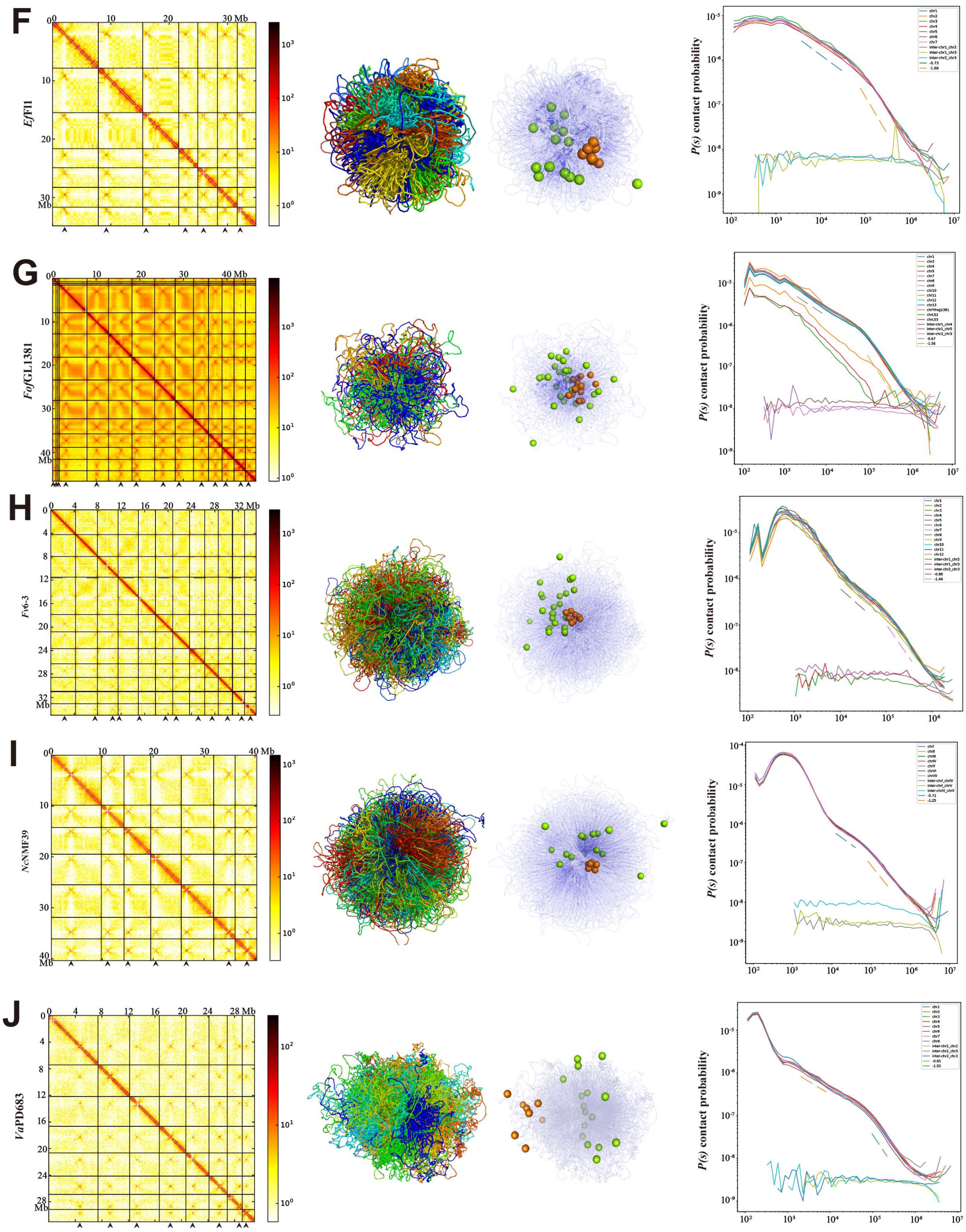

Extended Data Fig S3. *to be continued*

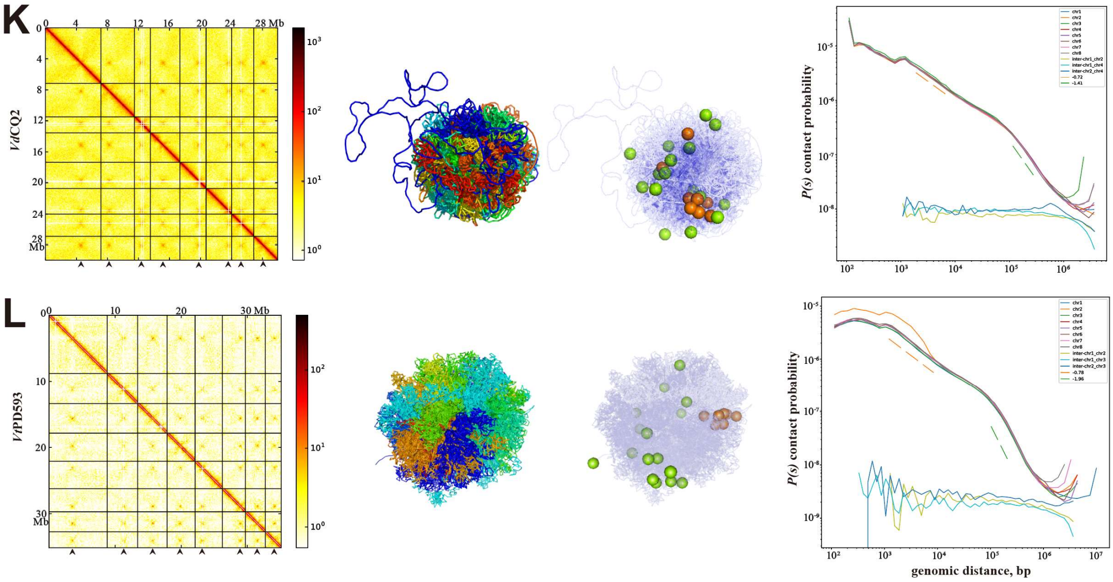

Extended Data Fig S3. Overview of 3D genome architecture of plant pathogenic fungi. Please refer to Figure 2 legend. Ab: *Agaricus bisporus*; Am: *Apiotrichum mycotoxinovorans*; Cfr: *Colletotrichum fructicola*; Ef: *Epichloe festucae*; Fof: *Fusarium oxysporum* f. sp. *fragariae*; Fv: *Flammulina velutipes*; Nc: *Neurospora crassa*; Pgt: *Puccinia graminis* f. sp. *tritici*; Pst: *P. striiformis* f. sp. *tritici*; Pt: *P. trinicina*; Va: *Verticillium alfalfa*; Vd: *Verticillium dahlia*; and Vt: *Verticillium tricorpus*. Please contact the first author for original high-quality figure.

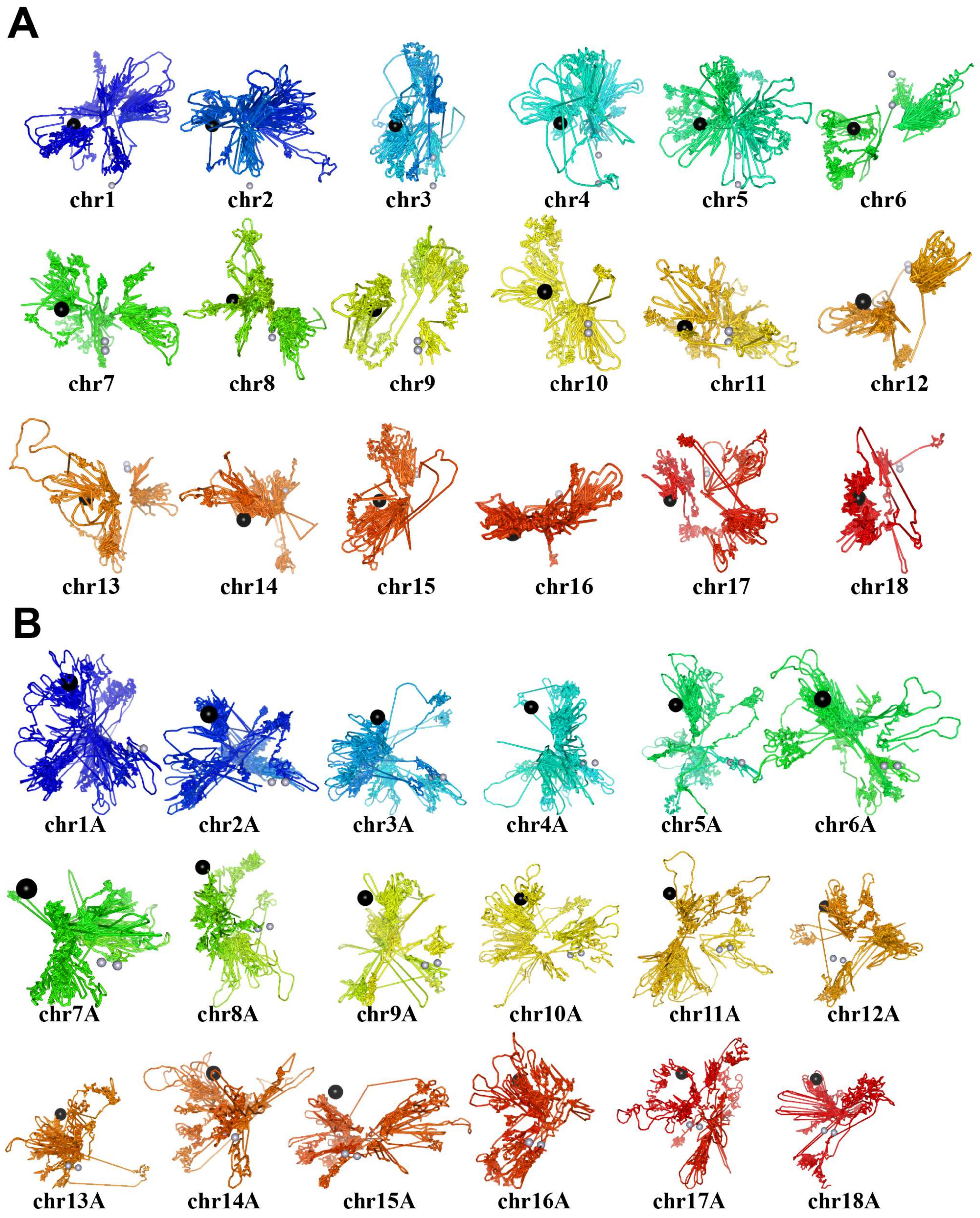

Extended Data Fig S4. 3D structure of separate chromosomes of CYR34 (a) and *Pgt*21-0 (b). The solid gray and black spheres represent the telomeres and centromeres, respectively.

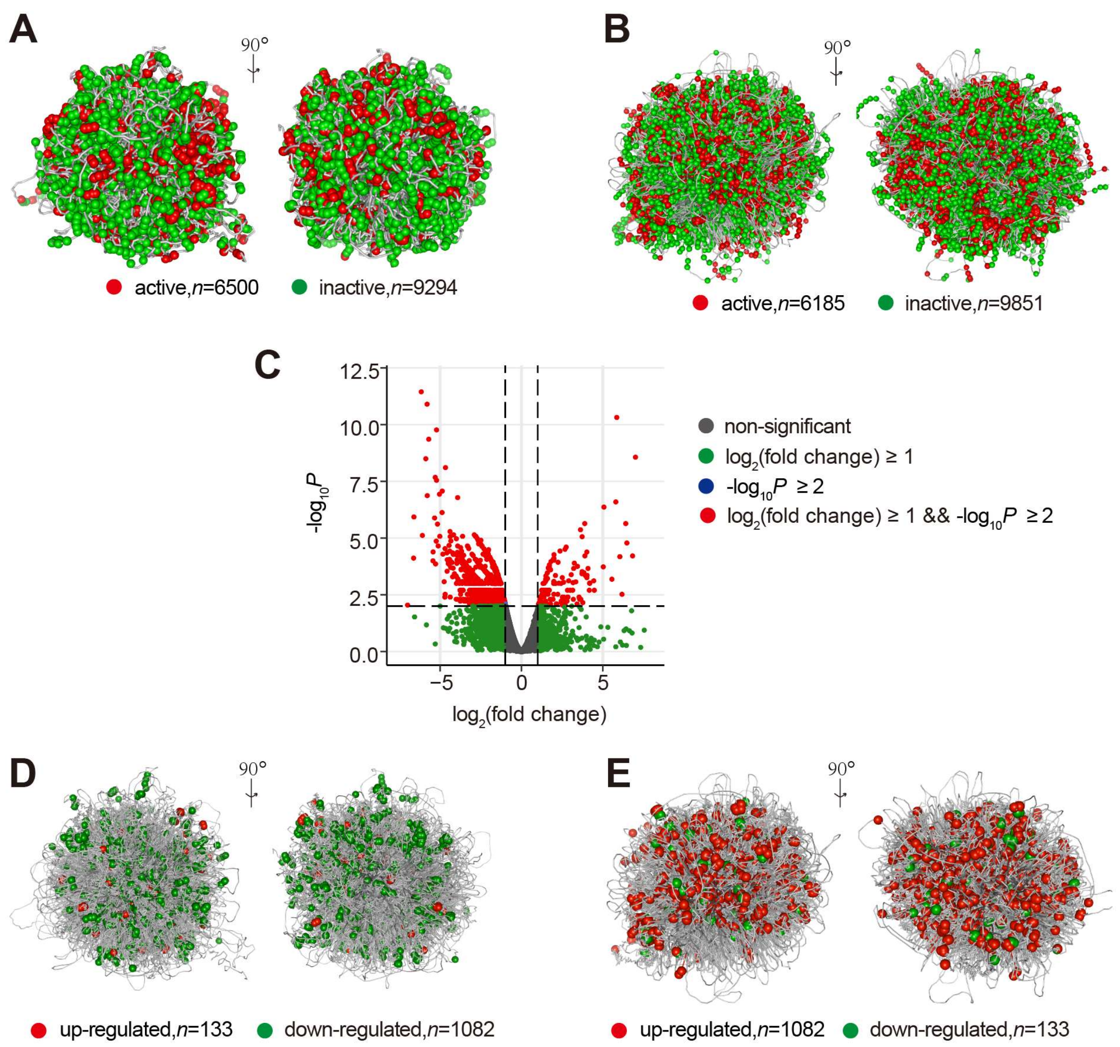

Extended Data Fig S5. 3D genome architecture and gene expression between urediniospore and germ-tube stages in CYR34. **(a-b)** The overview of the locations of active and inactive genes in the 3D genome, a: urediniospore; b: germtube. **(c)** The volcano plot of differentially expressed genes between urediniospore and germ-tube stages Expression difference with fold change≧2 and FDR corrected *P*≦0.01 was considered as significant. **(d-e)** The overview of the locations of up- and down-regulated genes in the 3D genome, d: rediniospore and e: germ-tube.

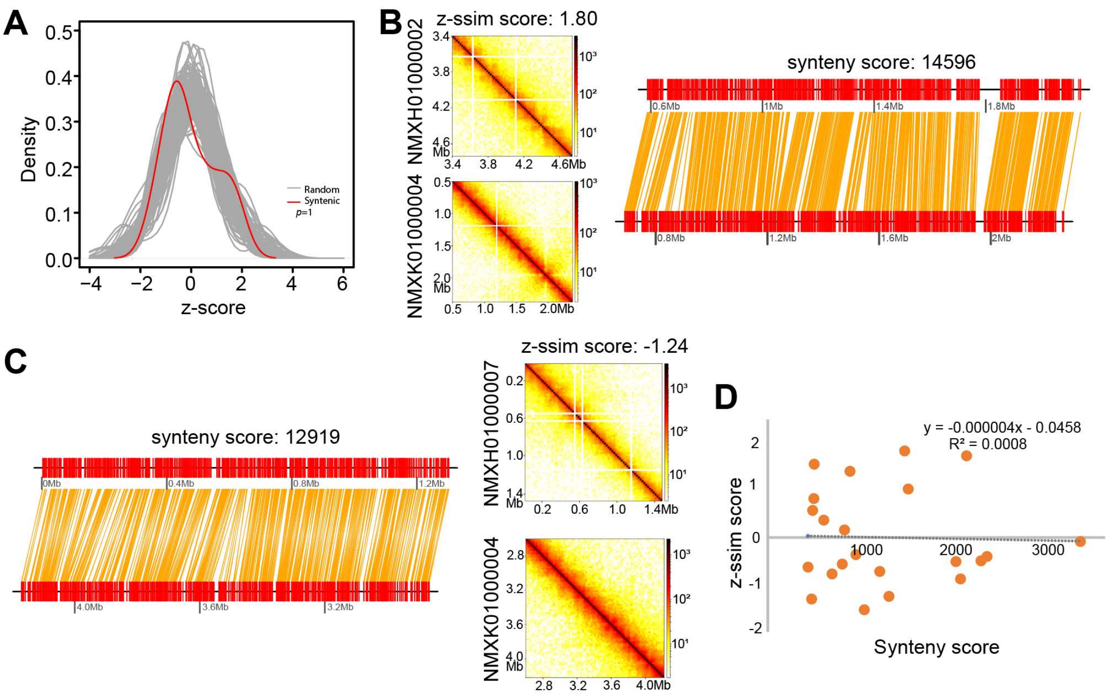

Extended Data Fig S6. Dynamics of chromatin conformational changes between *Verticillium alfalfae* and *V. tricorpus* syntenic regions. (a) Distributions of empirically determined CHESS z-scores for the syntenic region pairs in *V. alfalfae* (PD683) and *V. tricorpus* (PD593) (red line) and 100 random permutations of region pairs (gray lines). **(b)** and **(c)** Two examples of syntenic regions with different z-scores. **(d)** The distribution of the z-scores over syntenic scores.

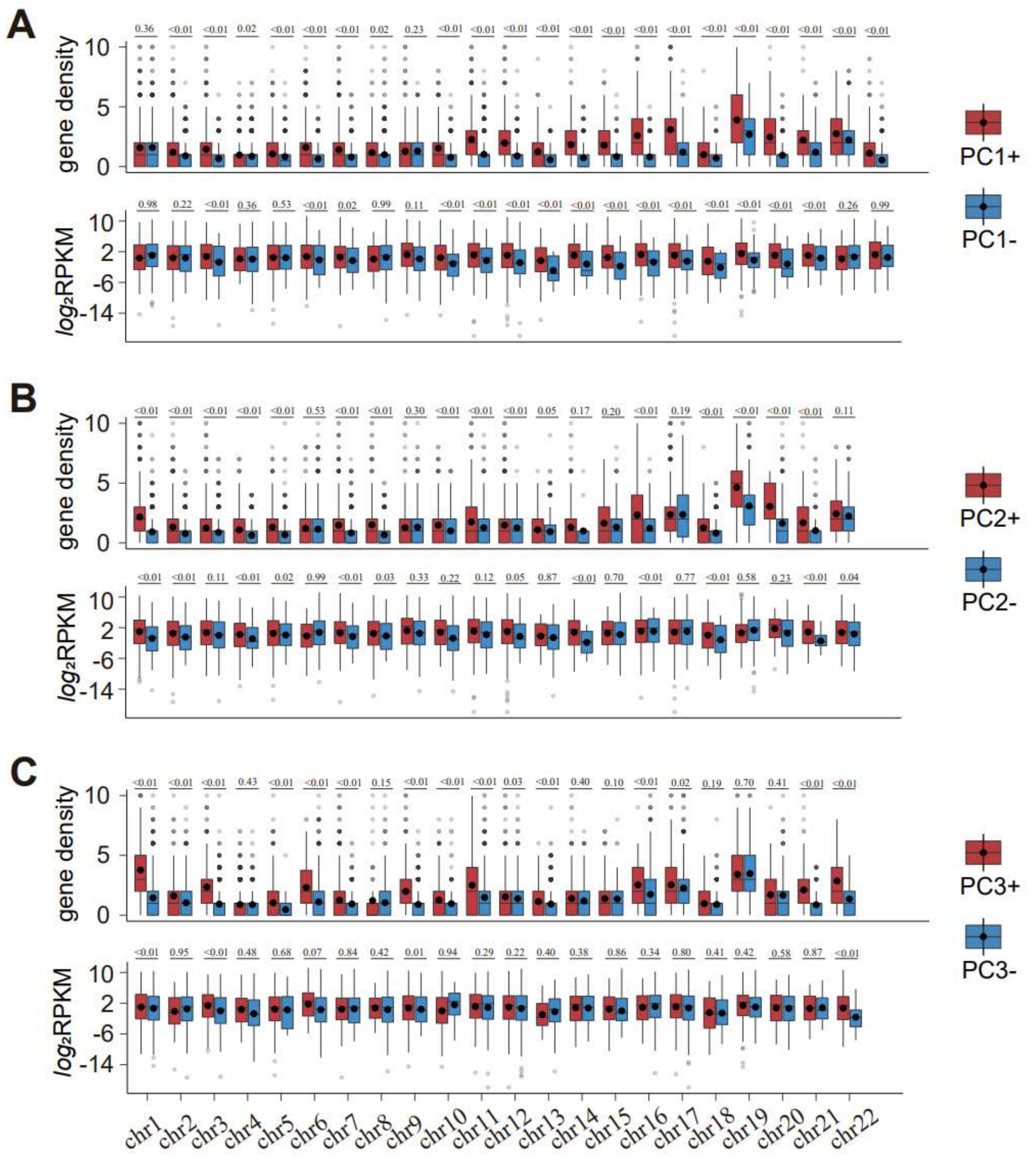

Extended Data Fig S7. Gene density and gene expression in PC+ and PC- regions in each chromosome in human Hap1-A cell. Please refer to Fig. 5. Note that for most chromosomes, the PC1'+' regions have significantly higher gene density and expression levels, corresponding to the 'A' compartment; while PC2 can separate the remaining chromosomes into compartments.

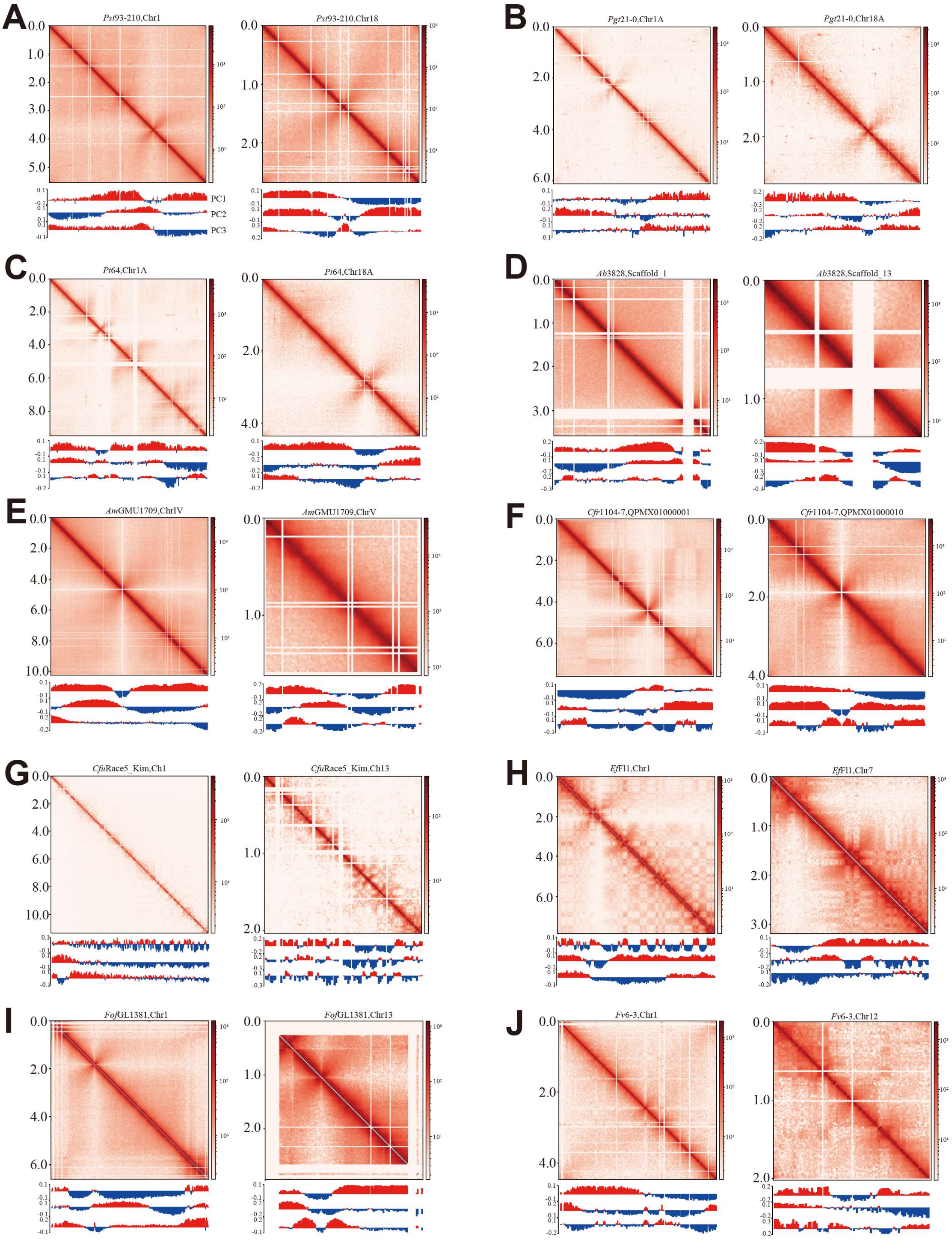

Extended Data Fig S8. *to be continued*

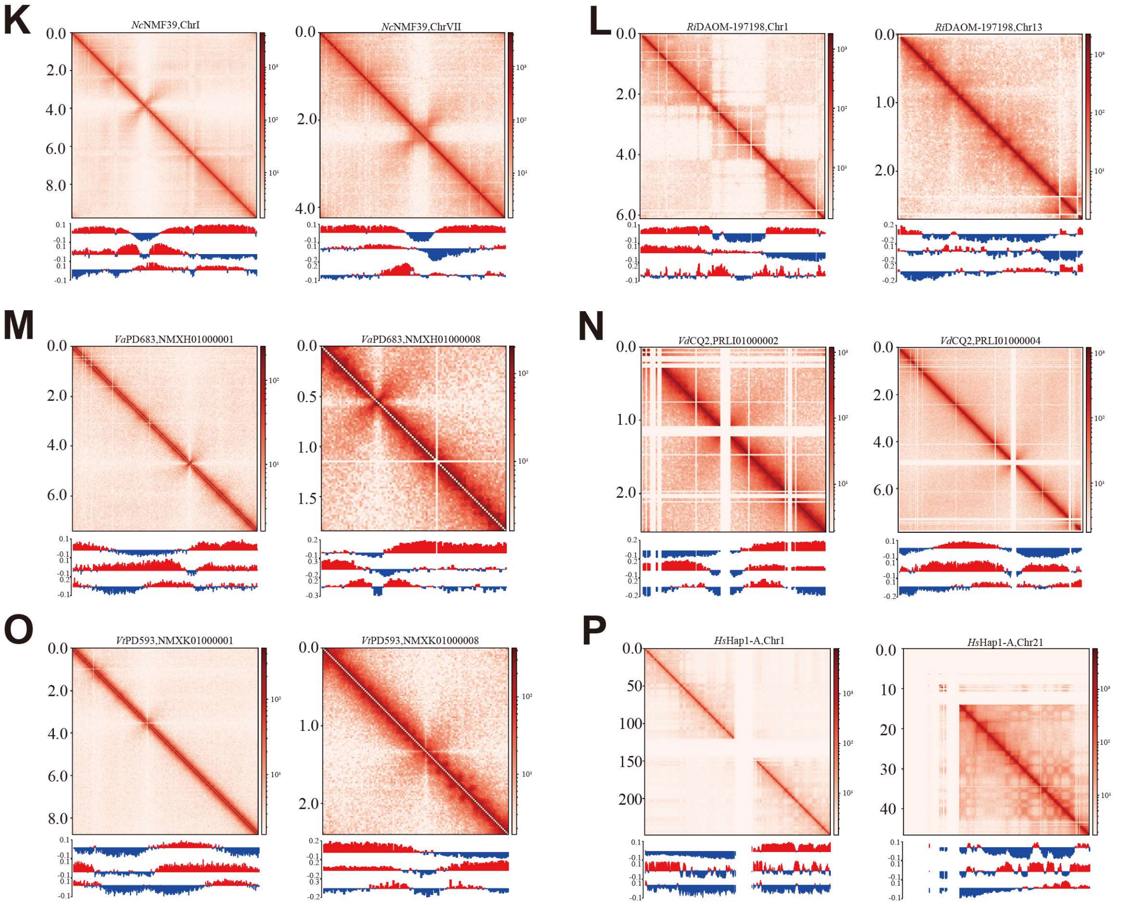

Extended Data Fig S8. Observed contact maps and distribution of first three PC eigenvectors in 15 fungi and human Hap1-A cells. For each organism only the longest and shortest chromosomes are plotted. Ab: *Agaricus bisporus*; Am: *Apiotrichum mycotoxinovorans*; Cfr: *Colletotrichum fructicola*; Cfu: *Cladosporium fulvum*; Ef: *Epichloe festucae*; Fof: *Fusarium oxysporum* f. sp. *fragariae*; Fv: *Flammulina velutipes*; Nc: *Neurospora crassa*; Pgt: *Puccinia graminis* f. sp. *tritici*; Pst: *P. striiformis* f. sp. *tritici*; Pt: *P. trinicina*; Ri: *Rhizophagus irregularis*; Va: *Verticillium alfalfa*; Vd: *Verticillium dahlia*; Vt: *Verticillium tricorpus*, and Hs: *Homo sapiens*.

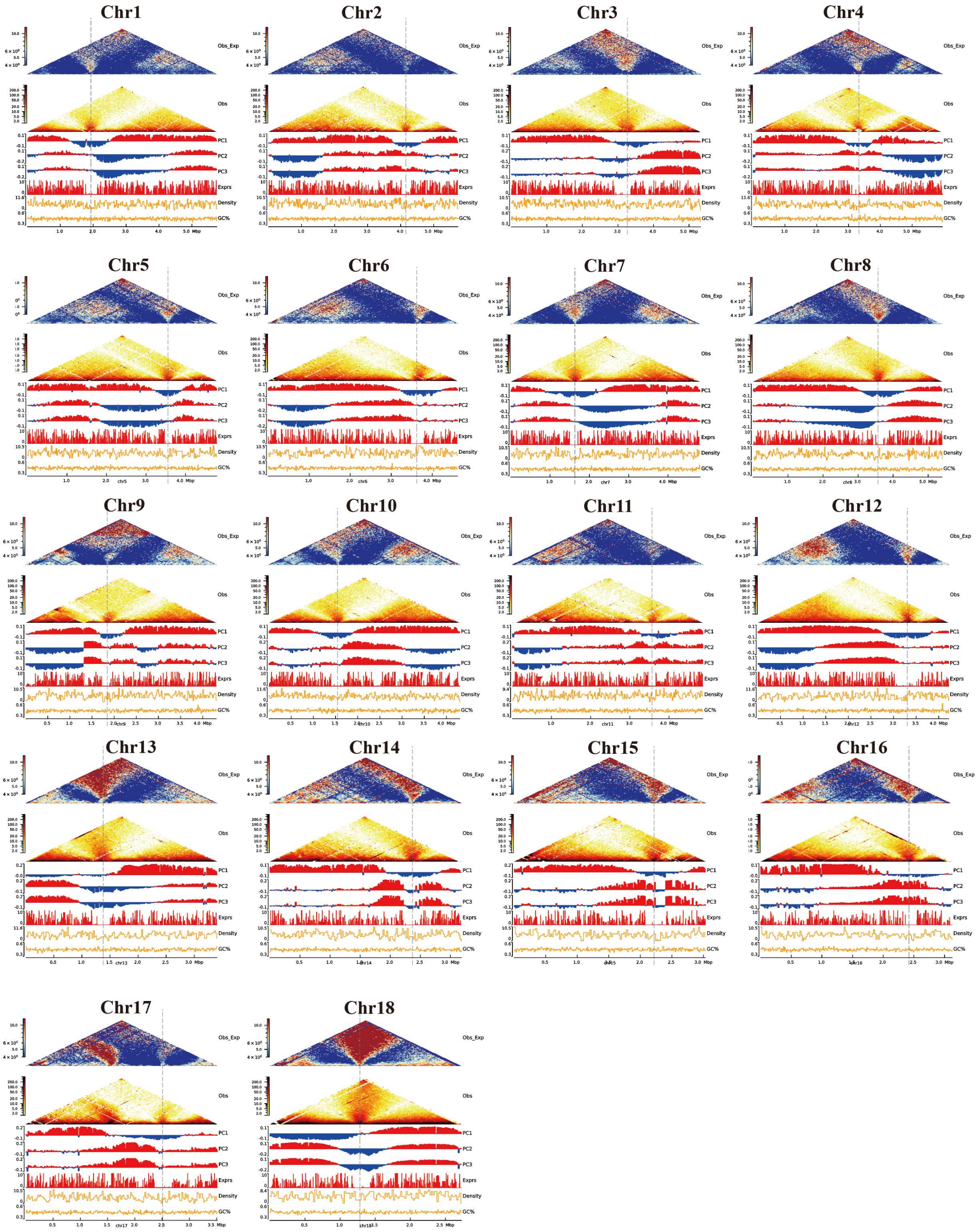

Extended Data Fig S9. Overview of 3D genome features of CYR34 individual chromosomes. For each chromosome, the first top track: heatmap of observed/expected contact matrix; second track: heatmap of observed contact matrix; the 3rd – 5th tracks: first three principal component eigenvalues; 6th track: log2 transformed gene express (in FPKM); 7th track: gene density (per 20kb); and 8th track: GC content. The centromeres were marked by the dashed vertical lines.

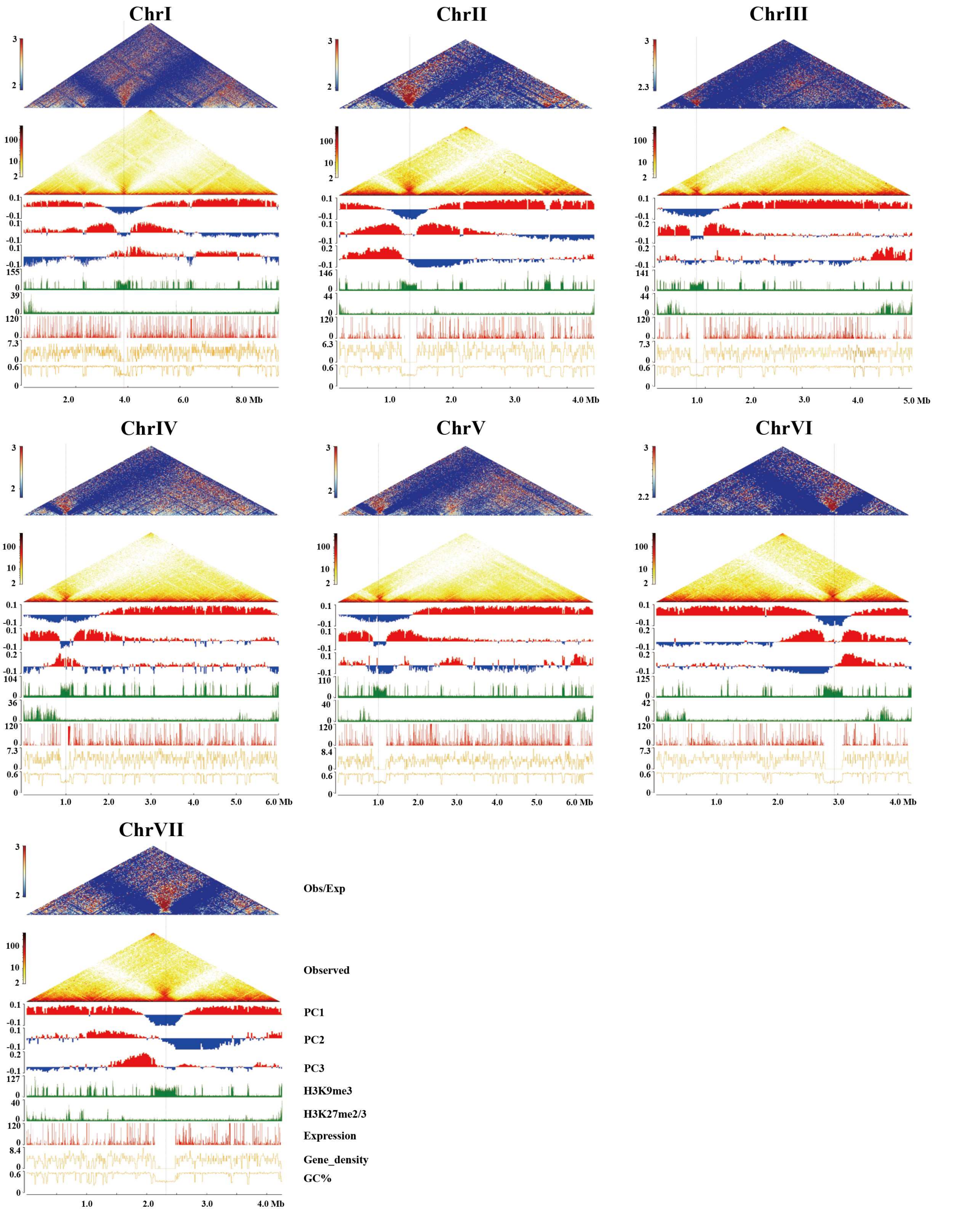

Extended Data Fig S10. Overview of 3D genome features of *Neurospora crassa* OR74A individual chromosomes. The tracks are same as in Supplementary Figure 9, except that the 6th track shows the peaks of H3K9me3, which is a epigenetic modification associated with transcriptionally silent heterochromatin, and the 7th track shows the peaks of H3K27me2/3, which is associated with gene repression.

Supplementary Table 1: Summary of data generated in this study.
Supplementary Table 2: Summary of CYR34 and *Pst*92-210 assemblies.
Supplementary Table 3: Summary of Hi-C data and valid contacts analyzed in this study
Supplementary Table 4a: Synteny regions and 3D genome similarity between *P. striiformis* f. sp. *tritici* CYR34 and *P. graminis* f. sp. *tritici* 21-0.
Supplementary Table 4b: Synteny regions and 3D genome similarity between *V. alfalfae* PD683 and *V. tricorpus* PD593.
Supplementary Table 5: Number of SMC (Structural Maintenance of Chromosomes) family proteins in the examined fungi.
